# Anticoagulant prescribing for atrial fibrillation and risk of incident dementia

**DOI:** 10.1136/heartjnl-2021-319672

**Published:** 2021-10-13

**Authors:** Sharon Louise Cadogan, Emma Powell, Kevin Wing, Angel Yun Wong, Liam Smeeth, Charlotte Warren-Gash

**Affiliations:** Department of Non-communicable Disease Epidemiology, London School of Hygiene & Tropical Medicine, London, UK

**Keywords:** atrial fibrillation, dementia, DOACs, vitamin K antagonists, electronic health records

## Abstract

**Objective:**

The aim of this study was to investigate the association between oral anticoagulant type (direct oral anticoagulants (DOACs) vs vitamin K antagonists (VKAs)) and incident dementia or mild cognitive impairment (MCI) among patients with newly diagnosed atrial fibrillation (AF).

**Methods:**

Using linked electronic health record (EHR) data from the Clinical Practice Research Datalink in the UK, we conducted a historical cohort study among first-time oral anticoagulant users with incident non-valvular AF diagnosed from 2012 to 2018. We compared the incidence of (1) clinically coded dementia and (2) MCI between patients prescribed VKAs and DOACs using Cox proportional hazards regression models, with age as the underlying timescale, accounting for calendar time and time on treatment, sociodemographic and lifestyle factors, clinical comorbidities and medications.

**Results:**

Of 39 200 first-time oral anticoagulant users (44.6% female, median age 76 years, IQR 68–83), 20 687 (53%) were prescribed a VKA and 18 513 (47%) a DOAC at baseline. Overall, 1258 patients (3.2%) had GP-recorded incident dementia, incidence rate 16.5 per 1000 person-years. DOAC treatment for AF was associated with a 16% reduction in dementia diagnosis compared with VKA treatment in the whole cohort (adjusted HR 0.84, 95% CI: 0.73 to 0.98) and with a 26% reduction in incident MCI (adjusted HR 0.74, 95% CI: 0.65 to 0.84). Findings were similar across various sensitivity analyses.

**Conclusions:**

Incident EHR-recorded dementia and MCI were less common among patients prescribed DOACs for new AF compared with those prescribed VKAs.

## Introduction

Atrial fibrillation (AF) is common among older people, with lifetime risk estimated at around 37% for people aged 55 years and over.^1^ AF is associated with adverse cardiovascular outcomes including a five-fold increase in stroke risk^2^ as well as incident heart failure, ischaemic heart disease and peripheral vascular disease.^3^ Dementia risk is approximately doubled among patients with AF^3–5^ and remains elevated by around 40% among individuals with no history of stroke. Putative mechanisms through which AF may influence dementia development are multifactorial and include ischaemic stroke or silent cerebral infarction, accumulation of microemboli, cerebral haemorrhage and systemic inflammation.^6 7^

Prophylactic oral anticoagulant (OAC) treatment is widely recommended to prevent stroke and systemic embolism in high-risk individuals with AF.^8^ A protective effect against various dementia subtypes is also plausible, given that cerebral hypoperfusion is intimately linked with neurodegeneration.^9^ A recent systematic review and meta-analysis of prospective studies (one RCT and five observational studies) showed a protective effect of OAC use compared with no OAC use against incident dementia in individuals with AF (RR 0.79 (95% C.I. 0.67 to 0.93)).^10^ Another study showed similar, though slightly attenuated, beneficial effects of OACs against a combined dementia and cognitive impairment outcome.^11^ For vitamin K antagonists (VKAs) such as warfarin, greater cognitive benefits were also seen with more time spent in therapeutic range. However, it was unclear whether different anticoagulant classes offered differing levels of cognitive protection. We hypothesised that direct oral anticoagulants (DOACs) such as apixaban may be more effective against incident dementia than VKAs due to better prevention of emboli, microvascular damage and strokes.^12^ However, existing evidence for such an effect is limited, especially among older populations with multiple comorbidities.

With the rising global burden of dementia due to population growth and ageing, and lack of effective treatments, dementia prevention is increasingly important. Understanding how, when and in whom to modify risk factors such as AF will inform dementia prevention strategies. We therefore aimed to investigate the association between DOACs or VKAs and incident dementia diagnoses in older patients newly diagnosed with AF using a longitudinal population-based cohort derived from linked primary and hospital care records from the UK.

## Methods

### Data sources

We used electronic health records (EHRs) from the UK Clinical Practice Research Datalink (CPRD) linked to Hospital Episodes Statistics (HES) and Index of Multiple Deprivation (IMD) data. CPRD Gold contains anonymised primary care records on diagnoses, tests, referrals, prescriptions and lifestyle factors collected during routine clinical care from practices using Vision software. Covering approximately 7% of the UK population, it is broadly representative in terms of age, sex and ethnicity.^13^ Around 80% of practices in England are linked to HES data, which comprise ICD-10 coded records of admissions to NHS hospitals in England since 1997. IMD data include area-based quintiles of deprivation based on patient or practice postcodes.

### Study design and population

We conducted a historical cohort study among individuals with an incident diagnosis of non-valvular AF at age 40 years or more, recorded in CPRD or HES between 01 January 2012 to 31 December 2018, with at least 12 months of research-standard CPRD registration. A visual presentation of the study design is shown in online supplemental figure 1. Patients were excluded if they had a history of dementia (mild cognitive impairment (MCI) for secondary analysis) or OAC prescription prior to AF (to prevent historical recording of anticoagulant use for other reasons). Patients with AF were identified in CPRD using Read codes and in HES using ICD-10 codes.

10.1136/heartjnl-2021-319672.supp1 Supplementary data

### Follow-up

Participants were followed from first OAC prescription until the earliest of: dementia diagnosis (MCI diagnosis for secondary analysis), death, transfer out of the general practice, last data collection date from the practice, end of OAC prescription or the end of study period (31 December 2018). For DOAC users, end of anticoagulant prescription was defined as last prescription date plus total days ‘on treatment’ (calculated using prescribed quantities and dosage information) plus an additional 30 days. For VKA (which has no fixed dose), we assumed that all days between two subsequent prescriptions were days on treatment, unless the gap between prescription dates exceeded 6 months or if there were no more prescriptions. In these cases, treatment was assumed to have stopped after 3 months.

### Definition of exposure and outcomes

Exposure (OAC type) was defined as the first record of OAC prescription, identified using product codes in primary care records and categorised into VKAs (warfarin, phenprocoumon, acenocoumarol) and DOACs (dabigatran etexilate, apixaban, rivaroxaban). Edoxaban was not included as it was licensed in the UK at the end of 2015 and is not currently included in NICE guidance for anticoagulation in AF, so the sample size would be small. We time-updated the exposure so that individuals who switched OAC type contributed exposed time initially to one class and then to the other.

Among VKA users, we also explored the role of time in therapeutic range (TTR) as a secondary exposure. Therapeutic range was defined as having an international normalised ratio (INR) between 2 and 3. INRs carried out during the initialisation period (within 30 days of first warfarin prescription) were excluded. After setting time zero as day 31 and, using INR values from months 1 to 6, the percentage of TTR was calculated using the Rosendaal method.^14^ Patients were classified as having ‘good control’ (TTR >70%), ‘intermediate control’ (TTR 50%–70%) and ‘poor control’ (TTR <50%).

The primary outcome was incident all-cause dementia, defined using Read codes for first clinical diagnosis of dementia in primary care records. In sensitivity analyses, we first expanded the outcome definition to include GP-recorded administrative codes as well as clinical codes to maximise sensitivity. Second, we restricted the sample to patients with linked secondary care data, which expanded the outcome to include incident dementia recorded in GP or hospital records. Third, to reduce the risk of reverse causality, we revised the outcome definition to include dementia occurring at least 1 year after first OAC prescription. Our secondary outcome was incident MCI, defined using clinical Read codes from primary care records.

### Covariates

The following demographic and lifestyle factors were also included (using data closest to first OAC prescription, where appropriate): sex (male/female), ethnicity (Black, White, South Asian, Mixed/Other) body mass index—calculated from height and weight if available, or as entered directly, practice level IMD in quintiles (quintile 1 being the least deprived), smoking status (current/non/ex-smoker), hazardous alcohol consumption (binary; identified using Read codes) and primary care consultation frequency in the year prior to first OAC prescription. We included the following clinical conditions recorded any time prior to OAC prescription: diabetes, hypertension, myocardial infarction, heart failure, stroke/thromboembolism, vascular disease, chronic renal disease and chronic liver disease. Other potential confounders, based on previous studies, included the following medications (recorded within 1 year prior to OAC prescription): statins, antiplatelet drugs or non-steroidal anti-inflammatory drugs, angiotensin-converting enzyme (ACE) inhibitor or angiotensin recepter blockers (ARB), beta-blockers, class 1 or 3 antiarrhythmics, digoxin, antipsychotics, antidepressants and proton pump inhibitors.

Codelists for all variables in the study are available on LSHTM Data Compass (DOI: https://doi.org/10.17037/DATA.00002326).

### Statistical analysis

Baseline characteristics were described by anticoagulant class (VKAs vs DOACS) for first OAC prescription. We also compared characteristics of patients who switched OAC type with those who did not. Crude incidence rates of dementia were calculated overall and separately for DOACs and VKAs. We generated cumulative incidence curves using the cumulative incidence function to describe dementia incidence over time by OAC group. Cox proportional hazards regression based on the cause-specific hazard was then used to calculate HRs for dementia among DOAC versus VKAs users, with deaths from competing risks censored.^15^ Age was used as the underlying timescale in the models to account for strong association between age and dementia.^16^ Stata’s ‘stsplit’ command was used to split records at time on OAC treatment (0–6, 6–12, 12–18, 18–24, 24–36, 36–48 and 48 plus months) and calendar year. First, we adjusted for age (as the time scale), calendar year, time on treatment and sex. We then added in socioeconomic/lifestyle factors, clinical conditions and medications in blocks. Results are presented for the final model adjusting for all co-variates, using complete case analysis.

Sensitivity analyses included (1) expanding the definition of dementia to include first clinical or administrative code for dementia, (2) repeating the analyses restricted to patients with linked primary care and hospital data, (3) only including dementia outcomes that occurred at least 1 year after first OAC prescription. In a secondary analysis, we also investigated the effect of OAC type on incident MCI. Finally, we conducted further analysis of the VKA sample to explore any association between TTR and incident dementia. All analyses were performed using Stata MP V.16 (StataCorp LP).

## Results

**Figure 1:**
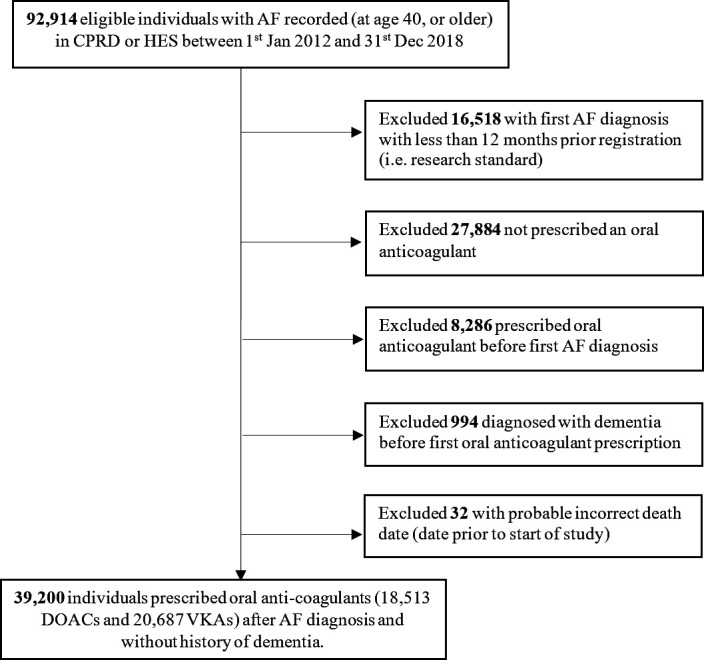
Flowchart of study participants. AF, atrial fibrillation; CPRD, Clinical Practice Research Datalink; DOACs, direct oral anticoagulants; HES, Hospital Episodes Statistics; VKA, vitamin K antagonists.

The study population comprised 39 200 individuals with incident AF diagnosed from 2012 to 2018, of whom 53% (N=20 687) were prescribed VKAs and 47% (N=18 513) DOACS at baseline, with 11% (N=4477) switching OACs during the study period (91% from VKAs to DOACs). Figure 1 shows the steps taken to identify eligible study participants and the final sample for this study.

Overall, 44.6% of the sample were female, with a median age of 76 years (IQR 68–83) and a median follow-up of 501 days (IQR 199–978). DOAC and VKA users were similar with respect to demographic and lifestyle factors, and there was no difference in the history of stroke, a major risk factor for dementia, between both groups. Nevertheless, patients prescribed VKAs generally had more comorbidities than those prescribed DOACs, particularly heart failure, other vascular disease and chronic renal disease. While patients prescribed DOACs were less likely to be using antiplatelet drugs (58.7% vs 68.7%), diuretics (43.5% vs 49.5%) and ACE inhibitors (37.8% vs 42.6%) compared with those prescribed VKAs at baseline, they had a greater use of beta-blockers (69.2% vs 66.0%), antidepressants (19.1% vs 17.7%) and proton pump inhibitors (46.7% vs 44.6%). Baseline characteristics of the sample by OAC type are shown in table 1. OAC treatment initiation patterns can be found in online supplemental figure 2. Characteristics of the 11% who switched OAC type during the study are shown in online supplemental table 1.

**Table 1:** Baseline characteristics of the sample, by first oral anticoagulant prescription

	VKA N=20 687	DOAC N=18 513
**Gender**		
Male	11 444 (55.3)	10 267 (55.5)
Female	9243 (44.7)	8246 (44.5)
**Age category, years**		
40–59	5854 (28.3)	5461 (29.5)
60–69	7821 (37.8)	6367 (34.4)
70–79	7012 (33.9)	6685 (36.1)
**Ethnicity**		
White	9930 (48.0)	8617 (46.5)
South Asian	122 (0.6)	82 (0.4)
Black	56 (0.3)	52 (0.3)
Mixed/other	92 (0.4)	65 (0.4)
Missing	10 487 (50.7)	9697 (52.4)
**Socioeconomic status (IMD—patient level**)		
1	2515 (12.2)	2395 (12.9)
2	2473 (12.0)	1881 (10.2)
3	2262 (10.9)	1738 (9.4)
4	1765 (8.5)	1278 (6.9)
5	1343 (6.5)	1051 (5.7)
Missing	10 329 (49.9)	10 170 (54.9)
**Socioeconomic status (IMD—practice level**)		
1	3774 (18.2)	3672 (19.8)
2	3337 (16.1)	2983 (16.1)
3	4622 (22.3)	4055 (21.9)
4	4242 (20.5)	3428 (18.5)
5	4712 (22.8)	4375 (23.6)
**BMI category**		
Underweight	418 (2.0)	414 (2.2)
Normal	5056 (24.4)	4727 (25.5)
Overweight	8372 (40.5)	7396 (40.0)
Obese	6841 (33.1)	5976 (32.3)
**Hazardous alcohol status**		
Yes	1245 (6.0)	1486 (8.0)
No	19 442 (94.0)	17 027 (92.0)
**Smoking status**		
Non/ex-smoker	18 679 (90.3)	16 419 (88.7)
Current	1981 (9.6)	2034 (11.0)
Missing	27 (0.1)	60 (0.3)
**Consultation frequency/year**		
1 to 10	2024 (12.2)	3393 (15.0)
11 to 20	5208 (31.4)	7075 (31.3)
21 to 30	4319 (26.0)	5342 (23.7)
31 to 40	2429 (14.6)	3084 (13.6)
41 to 50	1217 (7.3)	1658 (7.3)
51 to 60	588 (3.5)	838 (3.7)
Over 60	738 (4.4)	1155 (5.1)
None	92 (0.6)	40 (0.2)
**Calendar year of OAC prescription**		
2012	3975 (23.9)	170 (0.8)
2013	4456 (26.8)	1116 (4.9)
2014	3793 (22.8)	2618 (11.6)
2015	2401 (14.5)	4606 (20.4)
2016	1159 (7.0)	4839 (21.4)
2017	547 (3.3)	4805 (21.3)
2018	284 (1.7)	4431 (19.6)
**Conditions at baseline**		
Diabetes status	5538 (26.8)	4548 (24.6)
Hypertension history	14 317 (69.2)	12 403 (67.0)
Myocardial infarction history	2541 (12.3)	2071 (11.2)
Heart failure history	5225 (25.3)	3843 (20.8)
Stroke/transient ishemic attack/thromboembolism history	3776 (18.3)	3438 (18.6)
Vascular disease history	6002 (29.0)	4775 (25.8)
Renal disease history	6672 (32.3)	5235 (28.3)
Liver disease history	399 (1.9)	441 (2.4)
**Medications at baseline**		
Statin use	14 459 (69.9)	12 570 (67.9)
Antiplatelet drugs/NSAIDs	14 210 (68.7)	10 868 (58.7)
ACE inhibitors or ARBs	8806 (42.6)	7000 (37.8)
Beta-blockers	13 651 (66.0)	12 815 (69.2)
Class I or III antiarrhythmics	1461 (7.1)	1065 (5.8)
Digoxin	2524 (12.2)	1914 (10.3)
Diuretics	10 230 (49.5)	8045 (43.5)
Antidepressant medications	3667 (17.7)	3534 (19.1)
Antipsychotic medications	113 (0.5)	150 (0.8)
Proton pump inhibitors	9226 (44.6)	8644 (46.7)

ACE, angiotensin-converting enzyme; ARBs, angiotensin receptor blockers; BMI, body mass index; DOAC, direct oral anticoagulant; IMD, Index of Multiple Deprivation; NSAIDs, non-steroidal anti-inflammatory drugs; OAC, oral anticoagulant; VKA, vitamin K antagonist.

### Incidence rates of dementia and mild cognitive impairment

**Figure 2:**
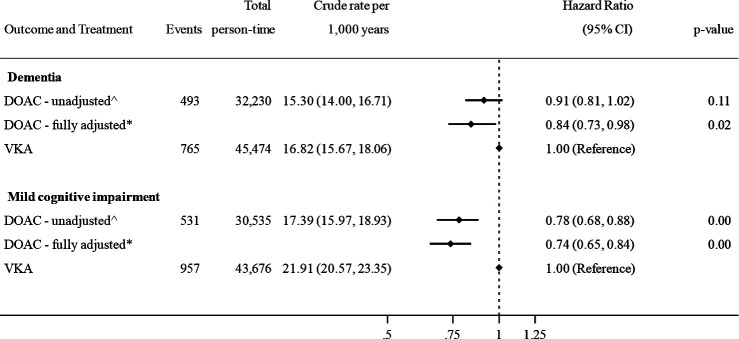
Association between oral anticoagulant use and incident dementia and mild cognitive impairment, defined using clinical codes. ˆAdjusted for age, calendar year, time-on-treatment and sex. *Adjusted for age, calendar year, time-on-treatment, sex, body mass index, smoking status, hazardous alcohol consumption, socio­economic status (practice level Index of Multiple Deprivation), primary care consultation frequency, diabetes, hypertension, myocardial infarction, statins, heart failure, stroke, vascular disease, renal disease, liver disease, antiplatelet drugs, ACE/ARB inhibitors, beta-blockers, antiarrhythmics, digoxin, diuretics, antipsychotics, antidepressants and proton pump inhibitors. DOAC, direct oral anticoagulant; VKA, vitamin K antagonist.

During follow-up, 1258 patients (3.2%) received a first-time diagnosis of GP-recorded dementia. The overall crude rate of all-cause dementia was 16.5 per 1000 person-years. The rate was slightly lower among patients prescribed DOACs compared with those prescribed VKAs (figure 2) and increased with age for both treatment groups (online supplemental figure 3). Overall, 1488 patients (4.0%) received a diagnosis of MCI, with a crude rate of 20.05 per 1000 person-years, which was lower among those prescribed DOACs (figure 2).

### DOACs versus VKAs and associations with dementia or MCI

Treatment of AF with a DOAC was associated with a 16% reduction in incident dementia diagnosis than treatment with a VKA (HR 0.84, 95% CI: 0.73 to 0.98), after adjusting for all covariates. DOACs were also found to be associated with a 26% reduction in MCI compared with VKAs (HR 0.74, 95% CI: 0.65 to 0.84). Age, sex and time-adjusted, and fully adjusted estimates are shown in figure 2. Partially and fully adjusted models are shown in detail in online supplemental table 2.

### Sensitivity analyses

Sensitivity analyses are shown in table 2. Results were similar when expanding the definition of dementia to first clinical or administrative code recorded in primary care records (N=39 028; HR 0.85, 95% CI: 0.72 to 1.01), although they just failed to reach statistical significance. A protective association was also seen when restricting the sample to patients with linked primary care and hospital records (N=18 080; HR 0.77, 95% CI: 0.60 to 0.99) and when only including dementia that occurred at least 1 year after first OAC prescription (N=39 200; HR 0.81, 95% CI: 0.67 to 0.98).

**Table 2:** Association between oral anticoagulant use and incident dementia, in a series of sensitivity analysis

	No of events	Total person-time (person-years)	Crude rate (per 1000 years)	Adjusted for age, calendar time, time on treatment and sex		Fully adjusted model*	
				HR (95% CI)	P value	HR (95% CI)	P value
(A) Dementia defined using clinical or administrative codes (N=39 028)*							
VKA	568	45 570	12.46 (11.48 to 13.53)	1.00		1.00	
DOAC	353	32 101	11.00 (9.91 to 12.21)	0.94 (0.79 to 1.11)	0.46	0.85 (0.72 to 1.01)	0.07
(B) Among patients with linked GP and hospital records (N=18 080)†							
VKA	319	19 205	16.61 (14.88 to 18.54)	1.00		1.00	
DOAC	173	12 907	13.40 (11.55 to 15.56)	0.84 (0.66 to 1.09)	0.19	0.77 (0.60 to 0.99)	0.04
(C) Dementia occurring at least 1 year after first oral anticoagulant prescription (N=39 200)*							
VKA	502	45 593	11.01 (10.09 to 12.02)	1.00		1.00	
DOAC	234	32 321	7.23 (6.37 to 8.23)	0.88 (0.73 to 1.07)	0.20	0.81 (0.67 to 0.98)	0.03

*Adjusted for calendar year, time-on-treatment, sex, body mass index, smoking status, hazardous alcohol consumption, socioeconomic status (practice level IMD), primary care consultation frequency, diabetes, hypertension, myocardial infarction, statins, heart failure, stroke, vascular disease, renal disease, liver disease, antiplatelet drugs, ACE/ARB inhibitors, beta-blockers, antiarrhythmics, digoxin, diuretics, antipsychotics, antidepressants and proton pump inhibitors.

†Adjusted for same covariates as above, except for socioeconomic status—patient-level IMD used for this model.

DOAC, direct oral anticoagulant; IMD, Index of Multiple Deprivation.

### Time in therapeutic range and incident dementia among VKA users

**Figure 3:**
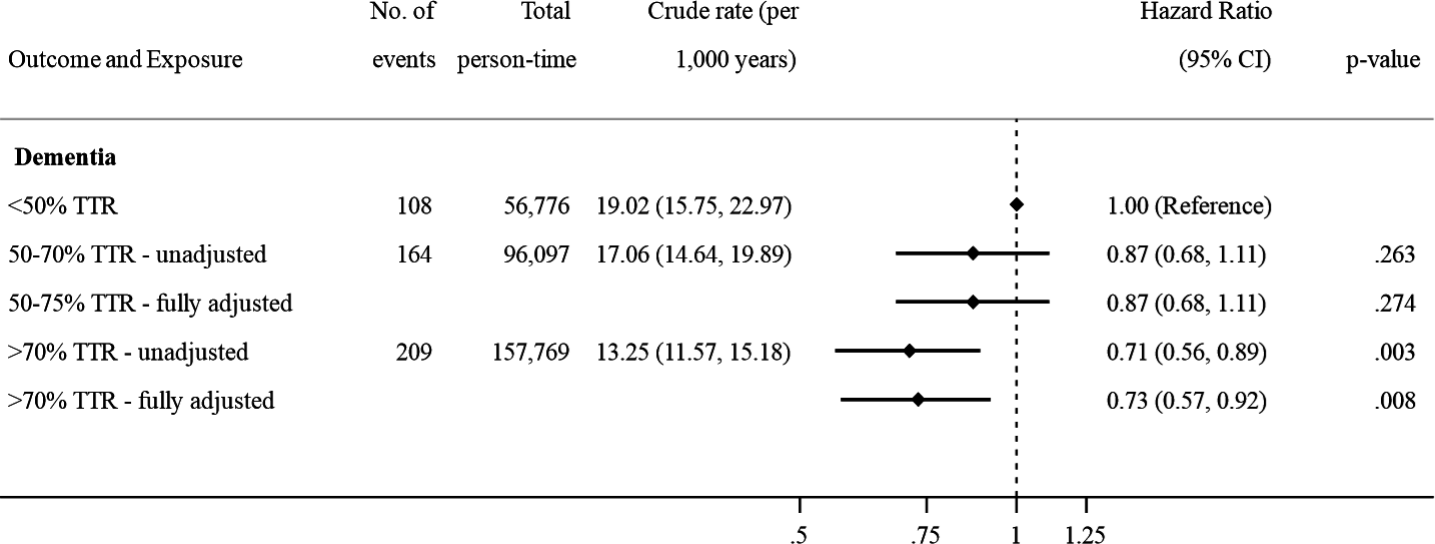
Association between time in therapeutic range (TTR) and risk of dementia among warfarin users (N=12 856). ˆAdjusted for age, calendar year, time-on-treatment and sex. *Adjusted for age, calendar year, time-on-treatment, sex, body mass index, smoking status, hazardous alcohol consumption, socio­economic status (practice level Index of Multiple Deprivation), primary care consultation frequency, diabetes, hypertension, myocardial infarction, statins, heart failure, stroke, vascular disease, renal disease, liver disease, antiplatelet drugs, ACE/ARB inhibitors, beta-blockers, antiarrhythmics, digoxin, diuretics, antipsychotics, antidepressants and proton pump inhibitors. Note: <50% TTR = poor INR control, 50–70 TTR = intermediate INR control, >70% TTR = good INR control. INR, international normalised ratio.

Among 12 856 VKA users with at least three INRs measured in months 1–6 (median number of INRs 12, IQR 9–15), 48% (N=6186) demonstrated good control (TTR >70%), 30.3% (N=3905) intermediate control (TTR 50%–70%) and 21.7% (N=2789) poor control (TTR <50%). Good INR control was associated with 27% reduction in incident dementia diagnosis (figure 3) compared with poor INR control (HR 0.73, 95% CI: 0.57 to 0.92).

## Discussion

In this population-based cohort study of 39 200 individuals, DOAC treatment for incident AF was associated with a 16% reduction in new diagnoses of all-cause dementia compared with treatment with VKAs. The magnitude and direction of effect estimates were consistent across sensitivity analyses. DOACs were also associated with a 26% reduction in incident MCI diagnoses.

Our results are consistent with studies suggesting favourable outcomes of DOACs compared with VKAs against composite vascular endpoints including dementia. A propensity score-matched cohort study of 5254 OAC users with AF from the USA showed that DOACs were associated with a reduction in stroke, transient ischaemic attack or dementia compared with warfarin use (HR 0.49, 95% CI 0.35 to 0.69).^17^ A large Swedish study comparing the effect of DOACs vs warfarin on ischaemic or haemorrhagic stroke or dementia among low-risk individuals suggested a protective effect of DOACs, although CIs were wide (HR 0.47, 95% CI 0.18 to 1.22).^18^ Although an earlier cohort study from Sweden showed no difference in dementia associated with DOACs compared with warfarin (HR 0.97, 95% CI 0.67 to 1.40), the study ended in 2014 and only 2.9% of the study population received a DOAC.^19^ While a recent study using UK primary care data found no significant difference in incident dementia or cognitive impairment in DOAC users versus warfarin (HR 0.89 95% CIs 0.70 to 1.14),^11^ as in our study, the direction of HRs favoured DOACs.

Prophylactic oral anticoagulation is recommended to prevent stroke among high-risk individuals with AF after taking bleeding risk into account.^8^ A systematic review and meta-analysis of trial data showed a 19% reduction in risk of stroke or systemic embolic events among participants receiving DOACs compared with warfarin.^20^ Similar results were seen in a large observational study using US claims data.^21^ While the protective association of DOACs that we showed against diagnosed dementia was smaller than effects identified in other studies, these may have been driven by the notable effectiveness of DOACs against stroke. Mechanisms to explain the link between AF and dementia include stroke, silent cerebral infarction and microemboli, as well as indirect effects of cerebrovascular hypoperfusion on oxidative stress, inflammation and blood–brain barrier disruption, which contribute to progression of both vascular dementia and Alzheimer’s disease.^22–24^

### Strengths and limitations

This is the first study using representative data from a real-world UK population to investigate the association between OAC type and dementia diagnosis. In contrast to other studies comparing the effect of OAC with no OAC, we used an active comparator new user design to reduce both confounding by indication and biases such as healthy user bias.^25^ In the UK, OACs for stroke prevention are not recommended to patients with AF aged under 65 years with low stroke risk (equating to CHA2DS2VASc score of 0 for men or 1 for women).^8^ We therefore did not include individuals without OACs who would have markedly lower stroke and dementia risk. In our sample, data on sociodemographic and lifestyle factors (excluding ethnicity) were >99% complete, enabling complete case analysis with negligible risk of bias. We carried out several sensitivity analyses to test the robustness of our assumptions.

There were however some limitations. While the positive predictive value of a dementia diagnosis in EHR data is over 80%,^26^ only two-thirds of people with dementia in the UK have their diagnosis recorded.^27^ The onset of dementia may lead to some individuals entering residential care. Although CPRD practices cover residential and nursing homes, some individuals may transfer to GP practices not included in CPRD for example, to be near family, which would result in missed diagnoses. As recording of dementia diagnosis may be more common among those who consult frequently, we adjusted for habitual consultation frequency. While warfarin users additionally require regular INR monitoring, all patients in our sample received regular prescriptions across the study period, so it is unlikely that dementia recording would differ by OAC type. As OAC data were obtained from prescriptions, it was not possible to assess medication adherence directly. A recent systematic review and meta-analysis suggests that one in three DOAC users has suboptimal adherence, taking their DOAC <80% of the time.^28^ In that study, pooled persistence was higher for DOACs than for VKAs. Differences in adherence may partly explain our findings: suboptimal adherence was associated with increased dementia risk among VKA users who spent <50% of time in the therapeutic range.

We allowed patients switch from one OAC class to another to reduce exposure misclassification that would have occurred increasingly over time if we had based exposure on first OAC prescription. We expected minimal carryover effects: effects of DOACs fade 12–24 hours after last dose; while warfarin may stay in the system for 3–4 days after last dose. In a study of a long-term outcome such as dementia, there would be minimal effect on exposure misclassification. While switching from a VKA to a fixed dose medication may be prompted by early cognitive problems, this would bias results towards the null, bringing rates of dementia among DOAC users closer to those of VKA users. In addition, our analysis of the secondary outcome MCI, which precludes the presence of functional impairment sufficient to prevent patients from managing a variable dose VKA, showed consistent results.

In common with other studies, VKA users had evidence of slightly more comorbidities than DOAC users,^29^ although history of stroke was similar between groups, and we controlled for a wide range of measured comorbidities as potential confounders. A previous study showed that patients with extensive cardiovascular comorbidities were less likely to have a dementia diagnosis in their primary care record, despite meeting standardised diagnostic criteria.^30^ If this were also true in our study, it suggests dementia might be under-recorded in VKA users, meaning we have underestimated the protective effect of DOACs. We did not have data on some dementia risk factors, such as history of traumatic brain injury or family history of dementia, although these are unlikely to affect anticoagulant choice. In future, alternative causal inference methods such as propensity scores or marginal structural models could be explored, to control for time-varying confounding. Investigating mechanisms through which DOACs may reduce dementia risk compared with VKAs should also be a focus of future research.

### Conclusions

We observed that individuals taking DOACs for AF were less likely to be diagnosed with dementia and MCI than those taking VKAs, after adjusting for potential confounding factors. While further evidence, including from randomised controlled trials, would strengthen this finding, it may be relevant to consider cognitive risk profile when prescribing OACs for AF among older individuals.

**Key messages**

**What is already known on this subject?**

- Oral anticoagulant use in atrial fibrillation (AF) has been linked with a reduction in dementia incidence.
- While direct oral anticoagulants (DOACs) offer superior protection against stroke and systemic embolism compared with vitamin K antagonists (VKAs) in randomised controlled trials (RCTs), it is unclear whether levels of cognitive protection differ by anticoagulant type.

**What might this study add?**

- We compared incident dementia diagnoses among individuals with new onset AF who were receiving either DOACs or VKAs in a large electronic health record-based cohort from the UK.
- DOAC treatment for incident AF was associated with a reduction in new diagnoses of all-cause dementia and mild cognitive impairment compared with treatment with VKAs.

**How might this impact on clinical practice?**

- It may be relevant to consider cognitive risk profile when prescribing oral anticoagulants for AF to older individuals.
- A better understanding of mechanisms through which anticoagulants influence dementia risk is needed, along with evidence of cognitive protection from RCTs.

10.1136/heartjnl-2021-319672.supp2 Supplementary data

## Data Availability

This study utilises data from the Clinical Practice Research Datalink, obtained under licence from the UK Medicines and healthcare products regulatory agency. The data is provided by patients and collected by the NHS as part of their care and support. The interpretation and conclusions contained in this study are those of the author/s alone. The data used in this study can only be used for the purposes set out in the submitted and approved ISAC protocol. no data can, therefore, be archived by the research team. Any future research would require a new application to CPRD with data obtained directly from CPRD, subject to their policies for scientific, data governance, and financial approvals (see www.cprd.com).

## References

[1] Staerk L , Wang B , Preis SR , et al . Lifetime risk of atrial fibrillation according to optimal, borderline, or elevated levels of risk factors: cohort study based on longitudinal data from the Framingham heart study. BMJ 2018;361:k1453. 10.1136/bmj.k1453 29699974 PMC5917175

[2] Wolf PA , Abbott RD , Kannel WB . Atrial fibrillation as an independent risk factor for stroke: the Framingham study. Stroke 1991;22:983–8. 10.1161/01.STR.22.8.983 1866765

[3] Kwok CS , Loke YK , Hale R , et al . Atrial fibrillation and incidence of dementia: a systematic review and meta-analysis. Neurology 2011;76:914–22. 10.1212/WNL.0b013e31820f2e38 21383328

[4] Kalantarian S , Stern TA , Mansour M , et al . Cognitive impairment associated with atrial fibrillation: a meta-analysis. Ann Intern Med 2013;158:338–46. 10.7326/0003-4819-158-5-201303050-00007 23460057 PMC4465526

[5] Santangeli P , Di Biase L , Bai R , et al . Atrial fibrillation and the risk of incident dementia: a meta-analysis. Heart Rhythm 2012;9:1761–8. 10.1016/j.hrthm.2012.07.026 22863685

[6] Ding M , Qiu C . Atrial fibrillation, cognitive decline, and dementia: an epidemiologic review. Curr Epidemiol Rep 2018;5:252–61. 10.1007/s40471-018-0159-7 30148041 PMC6096854

[7] Diener H-C , Hart RG , Koudstaal PJ , et al . Atrial fibrillation and cognitive function. J Am Coll Cardiol 2019;73:612–9. 10.1016/j.jacc.2018.10.077 30732716

[8] National Institute for Health and Care Excellence . Atrial fibrillation: management [Internet]. 2014 [cited 2019 Mar 19]. Report No.: CG 180. Available: https://www.nice.org.uk/guidance/CG180

[9] Di Marco LY , Venneri A , Farkas E , et al . Vascular dysfunction in the pathogenesis of Alzheimer's disease--A review of endothelium-mediated mechanisms and ensuing vicious circles. Neurobiol Dis 2015;82:593–606. 10.1016/j.nbd.2015.08.014 26311408

[10] Mongkhon P , Naser AY , Fanning L , et al . Oral anticoagulants and risk of dementia: a systematic review and meta-analysis of observational studies and randomized controlled trials. Neurosci Biobehav Rev 2019;96:1–9. 10.1016/j.neubiorev.2018.10.025 30391408

[11] Mongkhon P , Fanning L , Lau WCY , et al . Oral anticoagulant and reduced risk of dementia in patients with atrial fibrillation: a population-based cohort study. Heart Rhythm 2020;17:706–13. 10.1016/j.hrthm.2020.01.007 31931172

[12] Granger CB , Alexander JH , McMurray JJV , et al . Apixaban versus warfarin in patients with atrial fibrillation. N Engl J Med 2011;365:981–92. 10.1056/NEJMoa1107039 21870978

[13] Herrett E , Gallagher AM , Bhaskaran K , et al . Data resource profile: clinical practice research Datalink (CPRD). Int J Epidemiol 2015;44:827–36. 10.1093/ije/dyv098 26050254 PMC4521131

[14] Rosendaal FR , Cannegieter SC , van der Meer FJ , et al . A method to determine the optimal intensity of oral anticoagulant therapy. Thromb Haemost 1993;69:236–9. 10.1055/s-0038-1651587 8470047

[15] Lau B , Cole SR , Gange SJ . Competing risk regression models for epidemiologic data. Am J Epidemiol 2009;170:244–56. 10.1093/aje/kwp107 19494242 PMC2732996

[16] Canchola AJ , Stewart SL , Bernstein L . Cox regression using different timescales, 2003. Available: https://www.lexjansen.com/wuss/2003/DataAnalysis/i-cox_time_scales.pdf [Accessed 17 Aug 2021].

[17] Jacobs V , May HT , Bair TL , et al . Long-term population-based cerebral ischemic event and cognitive outcomes of direct oral anticoagulants compared with warfarin among long-term anticoagulated patients for atrial fibrillation. Am J Cardiol 2016;118:210–4. 10.1016/j.amjcard.2016.04.039 27236255

[18] Friberg L , Andersson T , Rosenqvist M . Less dementia and stroke in low-risk patients with atrial fibrillation taking oral anticoagulation. Eur Heart J 2019;40:2327–35. 10.1093/eurheartj/ehz304 31095295 PMC6642728

[19] Friberg L , Rosenqvist M . Less dementia with oral anticoagulation in atrial fibrillation. Eur Heart J 2018;39:453–60. 10.1093/eurheartj/ehx579 29077849

[20] Ruff CT , Giugliano RP , Braunwald E , et al . Comparison of the efficacy and safety of new oral anticoagulants with warfarin in patients with atrial fibrillation: a meta-analysis of randomised trials. Lancet 2014;383:955–62. 10.1016/S0140-6736(13)62343-0 24315724

[21] Lip GYH , Keshishian A , Li X , et al . Effectiveness and safety of oral anticoagulants among nonvalvular atrial fibrillation patients. Stroke 2018;49:2933–44. 10.1161/STROKEAHA.118.020232 30571400 PMC6257512

[22] Roher AE , Debbins JP , Malek-Ahmadi M , et al . Cerebral blood flow in Alzheimer's disease. Vasc Health Risk Manag 2012;8:599–611. 10.2147/VHRM.S34874 23109807 PMC3481957

[23] Tublin JM , Adelstein JM , del Monte F , et al . Getting to the heart of Alzheimer disease. Circ Res 2019;124:142–9. 10.1161/CIRCRESAHA.118.313563 30605407 PMC6319653

[24] O'Brien JT , Thomas A . Vascular dementia. Lancet 2015;386:1698–706. 10.1016/S0140-6736(15)00463-8 26595643

[25] Lund JL , Richardson DB , Stürmer T . The active comparator, new user study design in pharmacoepidemiology: historical foundations and contemporary application. Curr Epidemiol Rep 2015;2:221–8. 10.1007/s40471-015-0053-5 26954351 PMC4778958

[26] McGuinness LA , Warren-Gash C , Moorhouse LR , et al . The validity of dementia diagnoses in routinely collected electronic health records in the United Kingdom: a systematic review. Pharmacoepidemiol Drug Saf 2019;28:244–55. 10.1002/pds.4669 30667114 PMC6519035

[27] Alzheimer’s Research UK Dementia Statistics Hub . Dementia Diagnosis Rate [Internet]. 2018 [cited 2018 Jul 30]. Available: https://www.dementiastatistics.org/statistics/diagnoses-in-the-uk/

[28] Ozaki AF , Choi AS , Le QT , et al . Real-World adherence and persistence to direct oral anticoagulants in patients with atrial fibrillation: a systematic review and meta-analysis. Circ Cardiovasc Qual Outcomes 2020;13:e005969. 10.1161/CIRCOUTCOMES.119.005969 32148102

[29] Alamneh EA , Chalmers L , Bereznicki LR . The Tasmanian atrial fibrillation study: transition to direct oral anticoagulants 2011-2015. Cardiovasc Ther 2017;35:e12254. 10.1111/1755-5922.12254 28177198

[30] Aldus CF , Arthur A , Dennington-Price A . Undiagnosed dementia in primary care: a record linkage study [Internet]. Southampton (UK): NIHR Journals Library; 2020 [cited 2020 May 21]. (Health Services and Delivery Research). Available: http://www.ncbi.nlm.nih.gov/books/NBK555874/ 32310343

