# Supplementary material for: Anticoagulant prescribing for atrial fibrillation and risk of incident dementia
Source: Heart. 2021 Oct 13;107(23):1898–904. doi: 10.1136/heartjnl-2021-319672 (PMC8600601; doi:10.1136/heartjnl-2021-319672)
Supplement: Supplementary data [file heartjnl-2021-319672supp001.pdf]

Supplementary Table 1 Baseline characteristics of the sample, switchers versus non-switchers

	**Switchers**	**Non-switchers**
	4,485 (11.4%)	34,715 (88.6%)
**Gender**		
Male	2,320 (51.7)	19,391 (55.9)
Female	2,165 (48.3)	15,324 (44.1)
**Age category**		
40-49	85 (1.9)	630 (1.8)
50-59	317 (7.1)	2,366 (6.8)
60-69	998 (22.3)	6,919 (19.9)
70-79	1,647 (36.7)	12,541 (36.1)
>80	1,438 (32.1)	12,259 (35.3)
**Ethnicity**		
White	2,034 (45.4)	16,513 (47.6)
South Asian	22 (0.5)	182 (0.5)
Black	5 (0.1)	103 (0.3)
Mixed/Other	22 (0.5)	135 (0.4)
Missing	2,404 (53.6)	17,782 (51.2)
**Socio-economic status (IMD - patient level)**		
1	514 (11.5)	4,396 (12.7)
2	450 (10.0)	3,904 (11.2)
3	429 (9.6)	3,571 (10.3)
4	329 (7.3)	2,714 (7.8)
5	228 (5.1)	2,166 (6.2)
Missing	2,535 (56.5)	17,964 (51.8)
**Socio-economic status (IMD - practice level)**		
1	903 (20.1)	6,543 (18.8)
2	641 (14.3)	5,679 (16.4)
3	1,013 (22.6)	7,664 (22.1)
4	898 (20.0)	6,772 (19.5)
5	1,030 (23.0)	8,057 (23.2)
**BMI category**		
Underweight	99 (2.2)	733 (2.1)
Normal	1,129 (25.2)	8,654 (24.9)
Overweight	1,778 (39.6)	13,990 (40.3)
Obese	1,479 (33.0	11,338 (32.7)
**Hazardous alcohol status**		
Yes	4,109 (91.6)	32,360 (93.2)
No	376 (8.4)	2,355 (6.8)
**Smoking status**		
Non/ex-smoker	4,032 (89.9)	31,066 (89.5)
Current	451 (10.1)	3,564 (10.3)
Missing	2 (0.04)	85 (0.2)
**Consultation frequency/year^**		
1 to 10	476 (10.6)	4,941 (14.2)
11 to 20	1,321 (29.5)	10,962 (31.6)
21 to 30	1,145 (25.5)	8,516 (24.5)
31 to 40	723 (16.1)	4,790 (13.8)
41 to 50	377 (8.4)	2,498 (7.2)
51 to 60	171 (3.8)	1,255 (3.6)
Over 60	240 (5.4)	1,653 (4.8)

None	32 (0.7)	100 (0.3)
**Calendar year of OAC prescription***		
2012	811 (18.1)	4,103 (11.8)
2013	1,094 (16.1)	5,234 (15.1)
2014	1,036 (23.1)	5,767 (16.6)
2015	807 (18.0)	6,058 (17.5)
2016	466 (10.4)	5,064 (14.6)
2017	206 (4.6)	4,550 (13.1)
2018	65 (1.5)	3,939 (11.4)
**Conditions at baseline**		
Diabetes	1,165 (26.0)	8,921(25.7)
Hypertension history	3,176 (70.8)	23,544(67.8)
Myocardial infarction history	544 (12.1)	4,068 (11.7)
Heart failure history	1,221 (27.2)	7,847 (22.6)
Stroke/TIA/thromboembolism history	955 (21.3)	6,259 (18.0)
Vascular disease history	1,371 (30.6)	9,406 (27.1)
Renal disease history	1,485 (33.1)	10,422 (30.0)
Liver disease history	117 (2.6)	723 (2.1)
**Medications at baseline**		
Statin use	3,233 (72.1)	23,796 (68.6)
Antiplatelet drugs/NSAIDs	3,024 (67.4)	22,054 (63.5)
ACE inhibitor or ARB	1,826 (40.7)	13,980 (40.3)
Beta blockers	2,817 (62.8)	23,649 (68.1)
Class I or III antiarrhythmics	4,103 (91.5)	32,571 (93.8)
Digoxin	572 (12.8)	3,866 (11.1)
Diuretics	2,142 (47.8)	16,133 (46.5)
Antidepressants	945 (21.1)	6,256 (18.0)
Antipsychotics	33 (0.7)	230 (0.7)
Proton pump inhibitors	2,091 (46.6)	15,779 (45.5)

^Primary care consultations in the year prior to first oral anticoagulant prescription
*Year prescribed first oral anti-coagulant

Supplementary Figure 1: Graphical presentation of study design for primary analysis

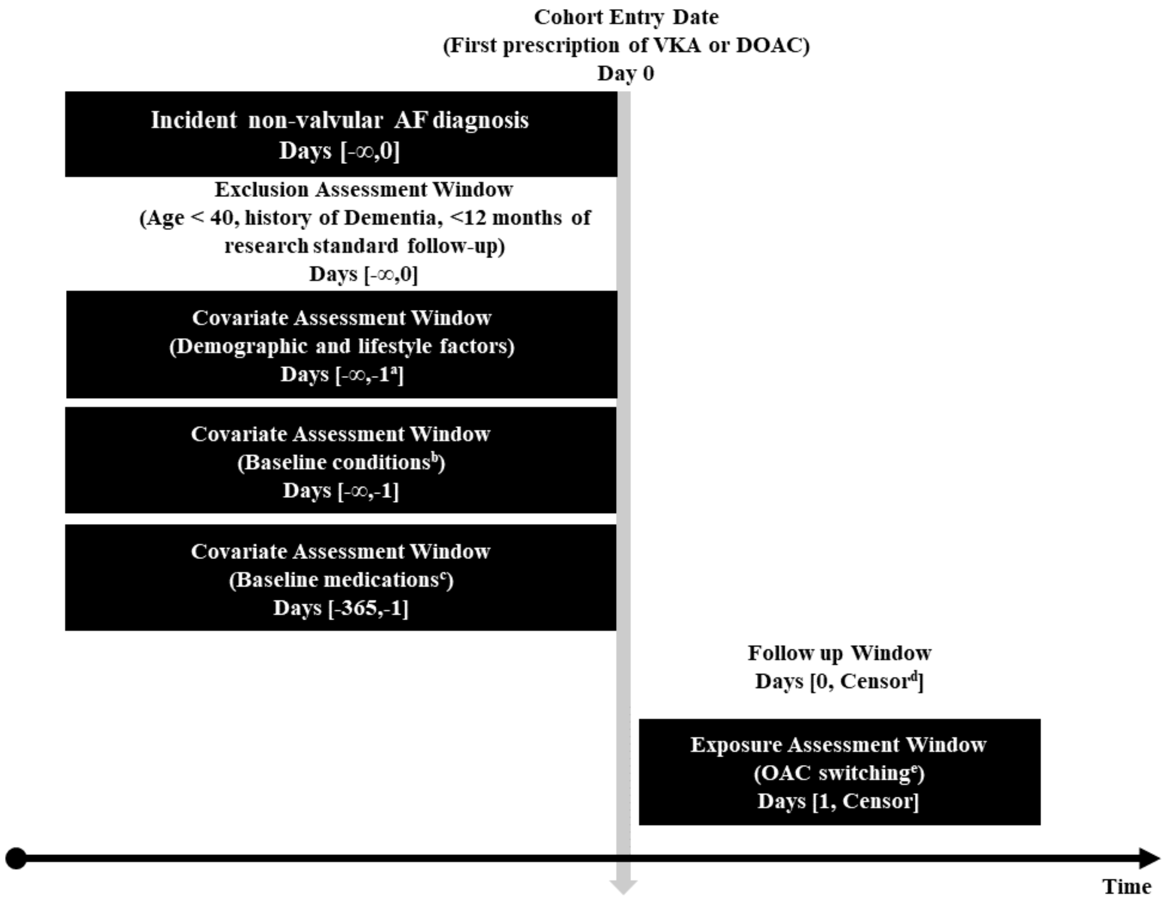

a. Demographic and lifestyle variables recording closest to first OAC prescription, where appropriate. Primary care consultation frequency in the year prior to first oral anticoagulant prescription.
b. Baseline conditions recorded at any time prior to OAC prescription: diabetes, hypertension, myocardial infarction, heart failure, stroke/ thromboembolism, vascular disease, chronic renal disease and chronic liver disease.
c. Medications recorded within one year prior to OAC prescription: statin use, antiplatelet drugs or non-steroidal anti-inflammatory drugs (NSAIDs), ACE inhibitor or ARB, beta blockers, class 1 or 3 antiarrhythmics, digoxin, antipsychotics, antidepressants, and proton pump inhibitors.
d. Censored at earliest of: dementia diagnosis, death, transfer out of the general practice, last data collection date from the practice, end of OAC prescription, or the end of study period (31 Dec 2018).
e. Patients initially prescribed one class of OAC, who then switched to the other class contributed exposure time initially to one class and then subsequently to the other.

Supplementary Figure 2: OAC treatment initiation among AF patients

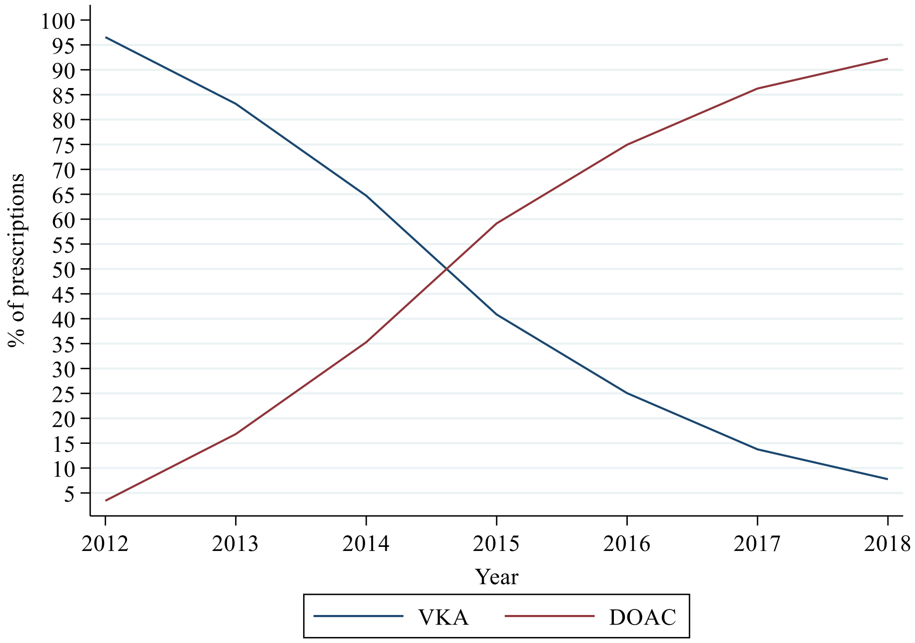

Caption: This figure illustrates the percentage of prescriptions for vitamin k antagonists (VKAs) versus direct oral anti-coagulants (DOACs), by year of prescription. Prescriptions represent the first prescription of DOAC and/or VKA per participant, to allow for switching.

Supplementary Figure 3: Cumulative incidence curves of dementia, by age

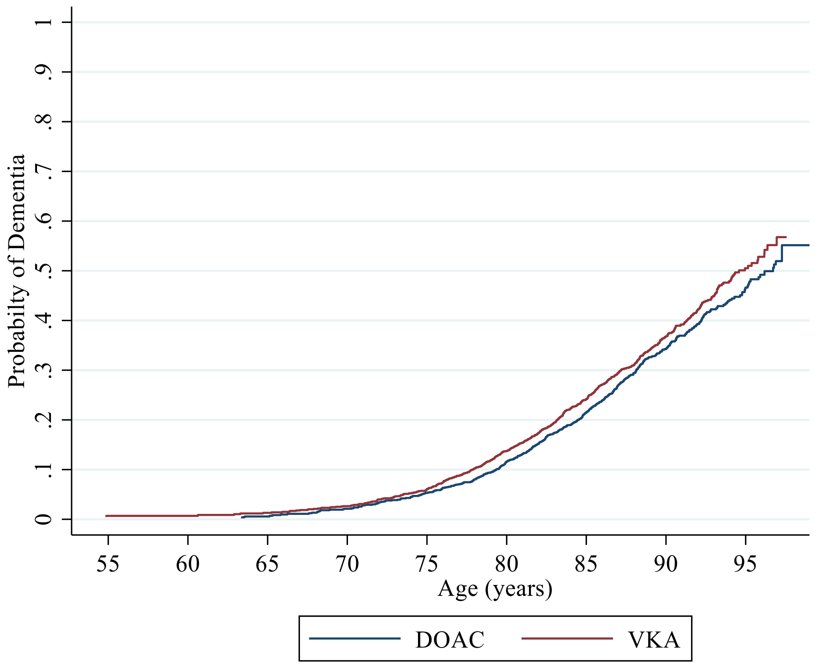

Caption: This figure shows the cumulative incidence of dementia (by age) in people with incident atrial fibrillation, treated with direct oral anti-coagulants versus vitamin k antagonists. The graph is censored at age 100 due to one outlier in the data.

Supplementary Table 2 Association between oral anticoagulant use and incident dementia defined using clinical codes – partially and fully adjusted primary analysis models

	**Adjusted for age, calendar year and time-on-treatment (as time-scales) and gender**				**Additionally, adjusted for demographic/lifestyle factors**				**Additionally, adjusted for demographic/lifestyle factors + clinical conditions**				**Additionally, adjusted for demographic/lifestyle factors + clinical conditions + medications**			
	HR	95% CI		p-value		95% CI		p-value	HR	95% CI		p-value	HR	95% CI		p-value
**OAC type**																
VKA					1.00				1.00				1.00			
DOAC	**0.92**	**0.79**	**1.06**	**0.25**	**0.90**	**0.78**	**1.04**	**0.14**	**0.87**	**0.75**	**1.00**	**0.05**	**0.84**	**0.73**	**0.98**	**0.02**
**Gender**																
Male	1.00				1.00				1.00							
Female	1.02	0.91	1.14	0.76	1.04	0.92	1.16	0.55	1.03	0.92	1.16	0.56	1.01	0.90	1.14	0.89
**BMI category**																
normal					1.00				1.00				1.00			
underweight					0.86	0.57	1.30	0.46	0.83	0.55	1.26	0.38	0.82	0.54	1.14	0.36
overweight					1.01	0.88	1.15	0.94	1.00	0.87	1.15	0.97	1.02	0.88	1.17	0.82
obese					1.00	0.86	1.16	0.97	0.99	0.85	1.15	0.89	1.02	0.87	1.19	0.83
**Smoking status**																
Non/ex-smoker					1.00				1.00				1.00			
Current					1.48	1.22	1.80	0.00[1]	1.46	1.19	1.78	0.00	1.43	1.17	1.74	0.00^
**Hazardous alcohol consumption**																
No					1.00				1.00				1.00			
Yes					1.23	0.97	1.57	0.08	1.23	0.97	1.57	0.09	1.20	0.94	1.54	0.14
**Socio-economic status - practice level IMD**																
1					1.00				1.00				1.00			
2					1.14	0.95	1.38	0.17	1.15	0.95	1.39	0.15	1.16	0.96	1.40	0.14
3					1.10	0.92	1.31	0.30	1.10	0.92	1.31	0.32	1.08	0.90	1.29	0.39
4					1.17	0.97	1.40	0.09	1.16	0.97	1.39	0.10	1.13	0.95	1.37	0.16
5					1.12	0.94	1.33	0.21	1.11	0.93	1.33	0.23	1.09	0.92	1.30	0.32
**Consultation frequency/year^**																
1 to 10					1.00				1.00				1.00			

11 to 20	0.99	0.81	1.21	0.94	0.98	0.80	1.20	0.85	0.96	0.78	1.18	0.70
21 to 30	1.06	0.86	1.30	0.59	1.04	0.85	1.28	0.71	1.02	0.82	1.25	0.89
31 to 40	1.19	0.95	1.48	0.13	1.17	0.93	1.46	0.18	1.13	0.90	1.42	0.31
41 to 50	1.47	1.14	1.88	0.00	1.41	1.10	1.81	0.01	1.32	1.02	1.71	0.04
51 to 60	1.15	0.83	1.61	0.40	1.11	0.80	1.55	0.54	1.03	0.73	1.45	0.85
over 60	1.51	1.14	2.02	0.00	1.46	1.09	1.95	0.01	1.37	1.02	1.86	0.04
**Clinical conditions**												
Diabetes					1.15	1.01	1.31	0.03	1.12	0.99	1.28	0.08
Hypertension					0.90	0.79	1.02	0.11	0.94	0.82	1.08	0.41
Myocardial Infarction					0.91	0.77	1.08	0.28	0.89	0.74	1.07	0.21
Heart failure					0.96	0.84	1.09	0.49	0.99	0.87	1.13	0.86
Stroke/TIA/thromboembolism					1.61	1.42	1.81	0.00	1.56	1.38	1.79	0.00
Vascular disease					1.09	0.97	1.22	0.16	1.08	0.96	1.22	0.18
Renal disease					1.04	0.92	1.17	0.56	1.05	0.93	1.19	0.41
Liver disease					0.92	0.57	1.49	0.73	0.90	0.54	1.43	0.60
**Medications**												
Antiplatelet/ NSAIDs									1.14	0.99	1.32	0.08
ACE inhibitors/ARB									1.10	0.97	1.24	0.13
Beta-blockers									1.04	0.92	1.17	0.51
Class I or III Antiarrhythmics									0.97	0.74	1.28	0.85
Digoxin									1.25	1.06	1.48	0.01
Diuretics									0.75	0.67	0.85	0.00
Antipsychotics									3.06	1.94	4.81	0.00
Antidepressants									1.53	1.33	1.75	0.00
Proton Pump Inhibitors									0.89	0.79	1.00	0.05
Statin use									0.97	0.87	1.13	0.62

^Primary care consultations in the year prior to first oral anticoagulant prescription. Note: ethnicity was not included in the final model due to the proportion of missing data.
