# Supplementary material for: Anticoagulant prescribing for atrial fibrillation and risk of incident dementia
Source: Heart. 2021 Oct 13;107(23):1898–904. doi: 10.1136/heartjnl-2021-319672 (PMC8600601; doi:10.1136/heartjnl-2021-319672)

Anticoagulant prescribing in atrial fibrillation (AF) and incident dementia

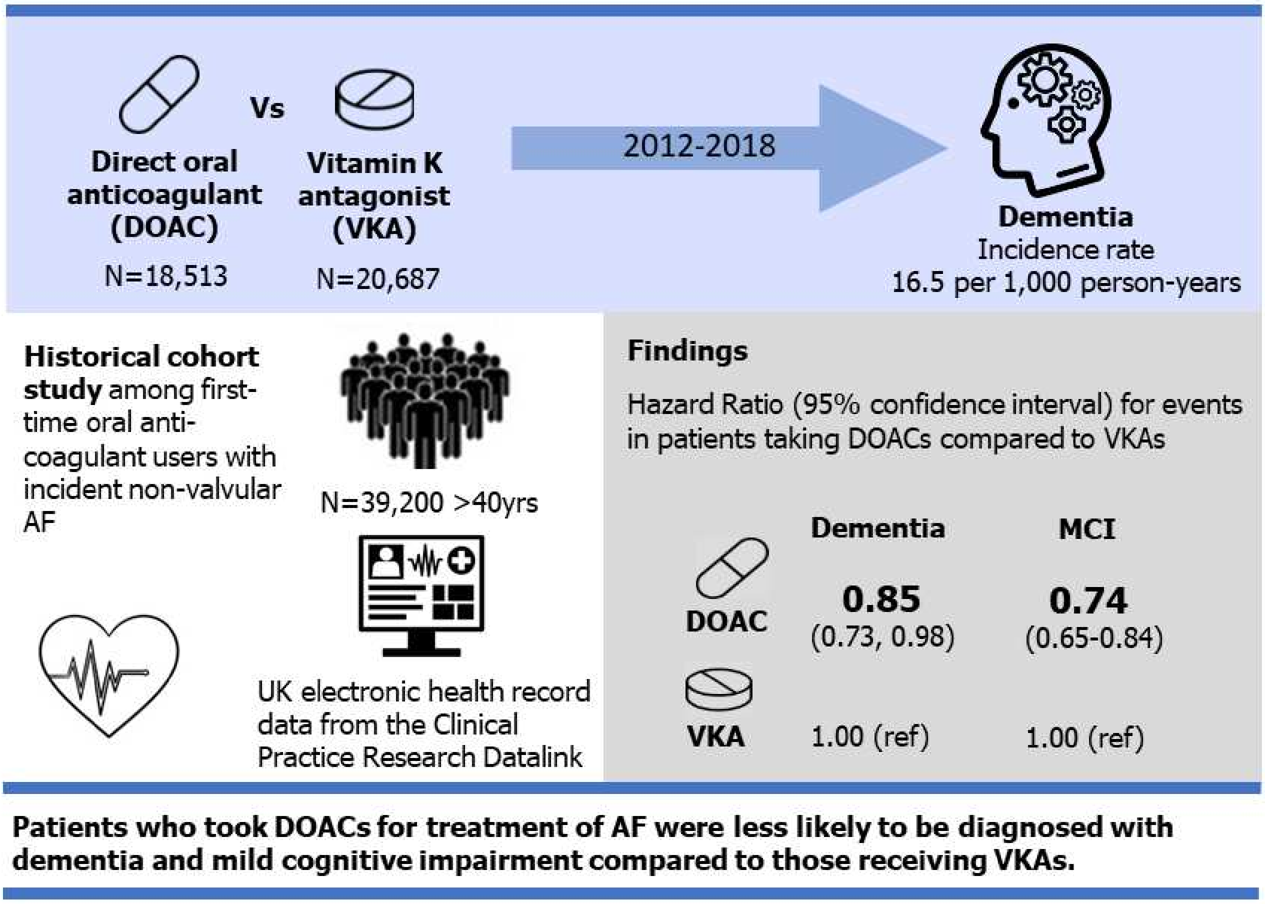

Supplement: Supplementary data [file heartjnl-2021-319672supp002.pdf]
